# Unraveling Mitral Annular Disjunction: A Case Report of Ventricular Arrhythmia Detected via Smartwatch

**DOI:** 10.3390/reports8020094

**Published:** 2025-06-14

**Authors:** Samantha Lo, Sanjay Sivalokanathan, Nina Kukar

**Affiliations:** 1 Icahn School of Medicine at Mount Sinai Elmhurst Hospital Center, Queens, NY 11373, USA; los2@nychhc.org; 2 Icahn School of Medicine at Mount Sinai Morningside/West, New York, NY 10025, USA; nina.kukar@mountsinai.org

**Keywords:** mitral annular disjunction, premature ventricular contractions, arrhythmia, cardiac MRI, sudden cardiac death

## Abstract

**Background and Clinical Significance:** Mitral valve prolapse (MVP) is commonly benign, but may result in life-threatening arrhythmias and sudden cardiac death (SCD). Mitral annular disjunction (MAD) often coexists with mitral valve prolapse (MVP) and has been implicated in the development of ventricular arrhythmias through myocardial stretch and fibrosis. **Case Presentation:** Here, we present a case that highlights the diagnostic value of multimodal imaging in evaluating ventricular ectopy in the context of MVP and MAD. A 72-year-old male presented to the cardiology clinic with palpitations and fatigue, compounded by an arrhythmia identified by his Apple Watch. Holter monitoring revealed premature ventricular contractions (PVCs), with cardiac magnetic resonance imaging (CMR) demonstrating MAD and basal inferolateral scarring. Despite minimal symptoms and normal echocardiographic imaging, CMR findings highlight the utility of advanced cardiovascular imaging in patients with newly detected ventricular arrhythmias. **Conclusion:** This case highlights the importance of integrating consumer wearables and advanced imaging in evaluating ventricular ectopy and its evolving role in risk stratification for patients with MVP, even in the absence of overt symptoms.

## 1. Introduction and Clinical Significance

Affecting up to 3% of the general population, mitral valve prolapse (MVP) is a common valve disorder characterized by myxomatous degeneration of mitral leaflets with displacement of one or both leaflets >2 mm superiorly into the left atrium during systole [1]. Symptoms of MVP may vary widely, with many patients reporting no symptoms, while others report symptoms such as palpitations, chest pain, and exertional dyspnea. A physical exam typically reveals a mid-systolic click followed by a late systolic murmur. In rare occurrences, patients with MVP may develop complications including mitral regurgitation, infective endocarditis, and malignant arrhythmias, which may lead to sudden cardiac death (SCD) [2].

Mitral annular disjunction (MAD) is a common finding in patients with MVP and is characterized by the atrial displacement of the mitral valve leaflet hinge, resulting in dissociation between the ventricular myocardium and the mitral annulus during systole. This displacement represents an area of thin fibrous tissue within the left ventricle implicated by recent studies in the development of ventricular arrhythmias. The correlation between SCD and MVP has been reported. However, the underlying mechanisms remain elusive. A systematic review of 79 articles describing 161 cases of MVP with SCD revealed increased propensity for young females with redundant bileaflet prolapse with SCD primarily attributable to ventricular arrhythmias [3]. It has been proposed that MAD is implicated in the development of ventricular arrhythmias, causing SCD in patients with MVP. However, the exact mechanism remains controversial. Importantly, the pathogenesis of SCD in MVP remains unclear. Through histological and cardiac magnetic resonance (CMR) studies, it is currently postulated that the mechanism by which MAD causes arrhythmias is due to the interplay between papillary muscle fibrosis and myocardial stretch [4]. In a study involving 116 patients with MAD, 22% of whom did not have MVP, Dejgaard et al. reported an increased risk of ventricular arrhythmias in patients who were younger, had a lower ejection fraction (EF), and had papillary muscle fibrosis. The authors report a high prevalence of severe arrhythmias in patients with MAD, with or without MVP, indicating MAD itself to be arrhythmogenic [5].

## 2. Case Presentation

**Figure 1:**
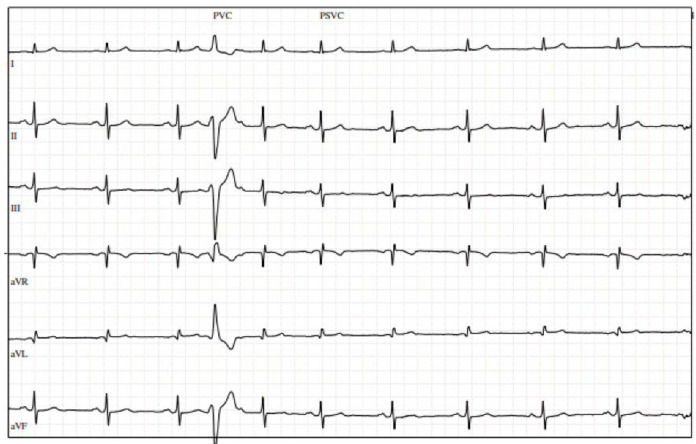
Stress electrocardiogram with premature ventricular contractions at rest.

A 72-year-old physically active gentleman with hyperlipidemia (HLD) was referred to cardiology after presenting with upper respiratory infection symptoms, fatigue, and episodes of atrial fibrillation (AF) detected by his Apple Watch. His vitals were notable for a heart rate of 55 bpm, with physical examination being unremarkable. Initial electrocardiogram (ECG) revealed a few premature ventricular contractions (PVCs). The patient was provided with a Holter monitor, which revealed a 13% (13,104/day) PVC burden, and underwent a transthoracic echocardiogram (TTE), which demonstrated a normal EF and mild left atrial dilation. Stress echocardiogram was negative for exercise-induced ischemia but revealed multiple PVCs at rest (Figure 1). 

**Figure 2:**
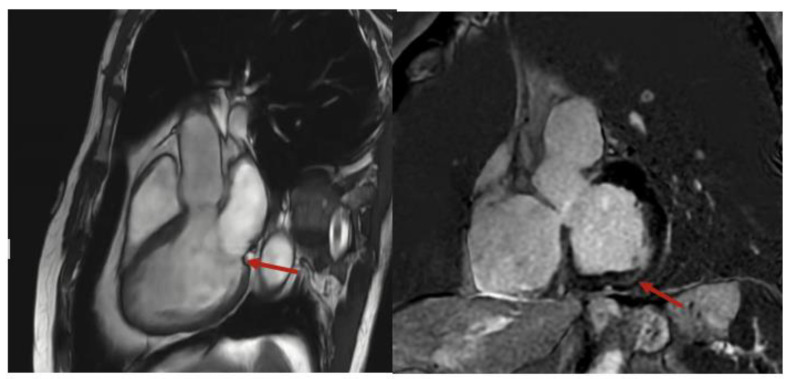
Cardiac magnetic resonance imaging with mitral annular disjunction in sagittal (**left**) and short axis (**right**) views. Red arrow indicates area of mitral annular disjunction and scarring, respectively.

He was referred to electrophysiology, started on metoprolol succinate, and underwent cardiac magnetic resonance (CMR), which demonstrated mild mitral annular disjunction (MAD), measuring 6 mm, with basal inferolateral left ventricular scarring (Figure 2), detected by late gadolinium enhancement (LGE). The patient was continued on metoprolol succinate, with a repeat Holter being organized to assess his PVC burden. He was referred to an electrophysiologist, but since his symptoms quiesced with a beta-blocker, neither an ablation nor implantation of a cardiac device was required. He remains asymptomatic, with no further episodes of tachycardia being recognized by his smart watch.

## 3. Discussion

The estimated prevalence of MVP is 1–3% in the general population, with a recent meta-analysis of 34 studies reporting an incidence of SCD in patients with MVP of 0.14% [6]. This represents a population at risk for SCD, the importance of which are reflected in the European Society of Cardiology’s (ESC) 2022 guidelines for management of patients with ventricular arrhythmias and prevention of SCD, where implantable cardioverter defibrillator (ICD) implantation is recommended for patients with valvular disease and persistent left ventricular dysfunction despite surgical intervention [7]. The mechanism by which arrhythmias develop in patients with MVP is unclear and elusive, although it is thought to involve structural abnormalities, such as leaflet redundancy and MAD, which can be demonstrated through echocardiography and CMR. Fortunately, advancements in imaging modalities have enabled the demonstration of a potential link between ventricular arrhythmogenicity, MVP, and MAD.

Interestingly, MAD may also be observed in normal hearts. A study utilizing CMR on a cohort of 98 subjects without valvular disease demonstrated the presence of MAD, defined as separation between the attachment of the mitral leaflet and the basal LV myocardium, in 96% of cases [8]. More importantly, the median height of disjunction was 3 mm in this normal cohort, compared to 5.2 to 10 mm in patients with MVP, and compared to 8 mm in patients with MVP and ventricular arrhythmias [9,10]. Taken together, these results suggest the height of MAD to be associated with arrhythmias. A 2020 analysis of 595 patients with MVP revealed that ventricular arrhythmias were associated with the male sex, severe mitral regurgitation (MR), the presence of more protracted displacement, and lower EF. Moreover, arrhythmia severity was positively correlated with mitral annular disjunction (MAD), mitral leaflet length and/or thickness, and left atrial and ventricular enlargement [10].

In patients with newly documented ventricular arrhythmias, the ESC guidelines strongly recommend obtaining an initial evaluation that includes a baseline ECG, a recording of the arrhythmia on ECG when possible, and an echocardiogram. ESC guidelines also recommend further evaluation, including CMR and a Holter monitor, in these patients with suspicion of structural heart disease, excluding coronary artery disease. A study examining cardiac pathology registries of patients with MVP and SCD found a high prevalence of left ventricular papillary muscle fibrosis (100%) and inferobasal wall fibrosis (88%) on histology, a finding similarly echoed using CMR on living patients with MVP and arrhythmias [11]. Importantly, scarring of the inferolateral basal myocardium, adjacent to the mitral annulus, is argued to correlate with ventricular arrhythmias and, consequently, SCD [4]. This fibrosis suggests that myocardial stretch by the prolapsing leaflet plays an arrhythmogenic role. Furthermore, these results underscore the potential utility of CMR in risk stratification and are reflected in the ESC’s recommendations, which suggest that CMR should be considered in the evaluation of patients presenting with newly documented ventricular arrhythmias and a suspicion of structural heart disease other than coronary artery disease [7]. While echocardiography is the mainstay of initial evaluations, CMR has become increasingly popular in the management of MVP. The advantages of CMR stem from its accurate characterization of dynamic and structural changes, such as MAD in living subjects, as well as its ability to detect myocardial fibrosis, both of which are linked to ventricular arrhythmias and SCD. Indeed, the use of CMR in the evaluation of patients with MVP has been on the rise, with recent work focusing on developing algorithms incorporating its use [12].

## 4. Conclusions

This case highlights the role of consumer wearable devices in detecting arrhythmias, prompting further evaluation, and the need for comprehensive assessment in patients with newly identified ventricular ectopy, even in asymptomatic patients. Advanced imaging, such as CMR, may uncover structural changes, such as mitral annular disjunction (MAD), which may predispose patients to arrhythmias and sudden cardiac death. Emerging research supports the utilization of CMR findings to risk-stratify and improve clinical decision-making in patients with mitral valve prolapse (MVP) and ventricular arrhythmias.

## Data Availability

The original contributions presented in this study are included in the article. Further inquiries can be directed to the corresponding author.

## Abbreviations

The following abbreviations are used in this manuscript:

AF	atrial fibrillation
CMR	cardiac magnetic resonance
ECG	electrocardiogram
EF	ejection fraction
HLD	hyperlipidemia
ICD	implantable cardioverter defibrillator
MAD	mitral annular disjunction
MR	mitral regurgitation
MVP	mitral valve prolapse
SCD	sudden cardiac death
TTE	transthoracic echocardiogram
